# Improved Prediction of Elastic Modulus for Carbon-Based Aerogels Using Power-Scaling Model

**DOI:** 10.3390/gels11030184

**Published:** 2025-03-06

**Authors:** Cheng Bi, Mingyang Yang, Xu Yang, Ke Yun, Yuan Lu, Ying Zhang, Jie Zheng, Mu Du

**Affiliations:** 1 Xi’an Special Equipment Inspection Institute, Xi’an 710065, China; 2 School of Resources Engineering, Xi’an University of Architecture and Technology, Xi’an 710055, China; 3 Xi’an Bright Laser Technologies Co., Ltd., Xi’an 710072, China; 4 School of Mechanical Engineering, Xi’an Shiyou University, Xi’an 710065, China; 5 Institute for Advanced Technology, Shandong University, Jinan 250100, China

**Keywords:** carbon-based aerogel, elastic modulus, thermal conductivity, power-scaling model

## Abstract

The mechanical stability of carbon aerogels, particularly their thermal insulation performance, is closely linked to their elastic modulus. This property plays a crucial role in determining the material’s overall mechanical stability. The objective of this study is to enhance the accuracy of elastic modulus predictions for carbon aerogels using a power-scaling model. By setting the prefactor of the Gibson and Ashby equation to 1.0, accurate predictions of the elastic modulus can be achieved if the correct scaling exponent is determined. Twelve sets of experimental data were used to fit the power-scaling model, revealing that the scaling exponent for the elastic modulus of carbon aerogels typically falls between 2.2 and 3.0. This range is narrower than the 2.0 to 4.0 range reported in the literature, with a median value of 2.6 providing reliable predictions. Additionally, a relationship between the solid thermal conductivity and the elastic modulus of carbon aerogels was established using a thermal conduction model. The study also examined the elastic modulus of carbon nanotube and graphene aerogels—both allotropes of carbon aerogel. By fitting experimental data into the power-scaling model, the scaling exponents for carbon nanotube aerogels and graphene aerogels were found to range from 2.7 to 3.5 and 2.7 to 3.7, respectively. Median exponent values of 3.1 and 3.2 were identified as optimal for predicting the elastic moduli of carbon nanotube and graphene aerogels.

## 1. Introduction

Like silica aerogels, carbon aerogels also exhibit low density, large specific surface area and high porosity, and their particular physical and chemical properties also make them multifunctional materials for many applications, such as electrochemistry, energy storage, adsorption, thermal insulation, catalysis, and medical treatment [1,2,3]. Among these usages, carbon aerogels are very worthy as super thermal insulation materials due to their low thermal conductivity and good thermal stability under non-oxidizing environments [4,5,6]. The low thermal conductivity of carbon aerogels can be attributed to its special nanoporous structure displaying a complex three-dimensional network skeleton consisting of random interconnected carbon nanoparticles, which can significantly suppress thermal conduction via the gas and solid phases of the material [7,8]. However, this structure cannot bond the carbon nanoparticles effectively, and results in poor mechanical properties, such as elastic modulus, compress strength, yield strength, and toughness [8,9,10]. Therefore, when carbon aerogels are used as thermal insulation material, analyses of the overall performance of the insulation material should consider both the thermal conductivity and mechanical properties, and the mechanical stability is the foundation for the non-failure of the thermal insulation material.

Many studies have focused on the heat-transfer mechanism in aerogels. Theoretical studies, experimental tests, and numerical simulations of thermal conductivity have been carried out, and accurate theoretical models of heat transfer in aerogels have been established, which can achieve optimal designs of the material and improve the performance of the thermal insulation [7,8,11,12,13,14]. To ensure the mechanical stability of aerogels, extensive efforts were devoted to investigating the mechanical properties to strengthen the mechanical stability of the aerogels, such as elastic modulus, compressive strength, yield strength, sound velocity, stiffness [3,5,8,15,16,17,18,19].

The elastic modulus of carbon aerogels reflects the ability of the resistance of the material to the elastic deformation, which is one of the most concerned mechanical properties when high stress concentration occurs in the material. Most of the above studies on the elastic modulus of carbon aerogels were experimental tests and numerical simulations, and the obtained elastic modulus data were relatively scattered, which cannot match the theoretical models well and cannot help in obtaining accurate predictions. Due to the convenience and simple calculation, the power-scaling model of porous material, which was proposed by Gibson and Ashby [20] in 1982, has been frequently used for calculating the mechanical properties of the aerogels, and can be used to predict many properties, such as elastic moduli, sound velocity, yield strength, collapse strength, and even thermal and electric conductivity [21]. However, for the elastic modulus of carbon aerogels, the scaling exponent in Gibson and Ashby’s formula is often distributed in a wide range, and may lead to significant deviations among the predictions. The wide range of the scaling exponent of the elastic modulus for carbon aerogels may be attributed to the following factors: (1) different ratios of sp2 to sp3 hybridized bonds in amorphous carbon lead to different properties under the same density [22,23,24], and (2) due to the brittleness of carbon aerogels, the mechanical experiment may easily cause damage to the material, which will result in fluctuations in the tests of the elastic modulus. Therefore, it is necessary to obtain an accurate scaling exponent as well as a narrow distribution range to improve the prediction accuracy of the power-scaling model, and finally provide theoretical guidance for the design of mechanical properties of carbon aerogels.

The aim of this work is to narrow the scaling exponent range of the carbon aerogel elastic modulus, and then improve the prediction accuracy of the power-scaling model. In this study, the scaling exponent range of the carbon aerogel elastic modulus is obtained by the fittings of the experimental data, and the data are employed in two ways: (1) we directly cite open experimental data in the literature (mechanical experiments), which may contain some “destructive” factors, and (2) indirectly convert from the experimental values of the thermal conductivity, where the conversion method can be regarded as a “non-destructive” test method for the aerogel elastic modulus proposed in the authors’ previous work [25]. By comparing and analyzing the results of the two groups of data, we can obtain the expected results. In addition, carbon nanotube and graphene are the two most interesting carbon allotropes, and the corresponding aerogels named carbon nanotube aerogel and graphene aerogel also belong to the class of the carbon aerogels, so the elastic modulus of these two types of carbon aerogels will be also discussed in this study. For convenience, the carbon aerogel in this study specially denotes the traditional aerogel, i.e., amorphous porous material of carbon particle assembly.

## 2. Results and Discussion

### 2.1. Validation of Thermal Conduction Model of Elastic Modulus of Carbon Aerogels

**Figure 1:**
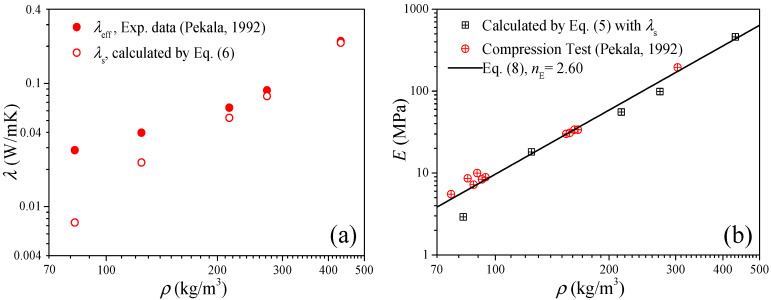
Validation of the thermal conduction model of the elastic modulus of Equation (5). (**a**) Thermal conductivity as the input parameters. (**b**) Elastic modulus as the results (room temperature).

In this section, three groups of available data of carbon aerogels are employed to validate the thermal conduction model of elastic modulus. The first case is to examine Equation (5) by the data of the “R/C = 200” carbon aerogels in Ref. [26]. Figure 1a shows the measured data of the effective thermal conductivity (solid symbols) and the solid thermal conductivity (open symbols) of the carbon aerogels, where the solid thermal conductivity as the input parameter of Equation (5) was extracted from the effective thermal conductivity by the heat transfer model of Equation (6).

Figure 1b shows the comparison between the experimental data and the predictions of Equation (5), and good agreements can be observed as *ρ* > 120 kg/m^3^. For *ρ* < 100 kg/m^3^, we can find some large deviations between the experimental data and predictions from Equation (5), and these deviations may be attributed to two factors: (1) for low-density aerogels, the compression test may easily cause some damage to the material, which will lead to a few test fluctuations; and (2) since the elastic modulus depends on the bulk sound velocity and density, for low-density carbon aerogel (atmospheric pressure), the air density will be not neglected compared with the carbon aerogel, and the sound velocity is lower than that of the air (about 340 m/s); therefore, the elasticity of the air will have a certain impact on the elasticity test [27]. In addition, the solid line in Figure 1b is fitted by Equation (8) based on the experimental data and predictions (discussed in Section 2.2), and under the guidance of this straight line, we can believe that the solid thermal conductivity can obtain accurate predictions for the elastic modulus of the carbon aerogels.

**Figure 2:**
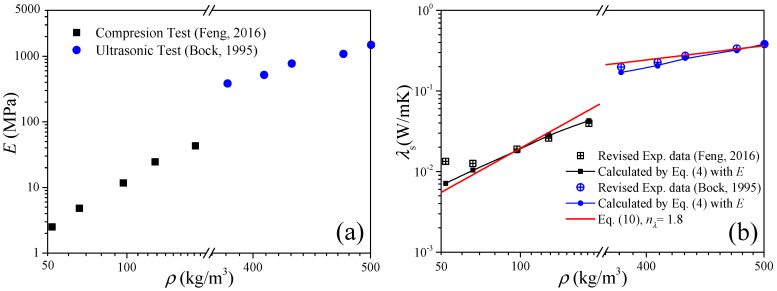
Validation of the thermal conduction model of elastic modulus of Equation (4). (**a**) Elastic modulus as the input parameter. (**b**) Solid thermal conductivity as the results (room temperature).

The second example is to validate Equation (4) with the data of low-density and high-density carbon aerogels published in Refs. [5,28], respectively. In this case, the elastic modulus is the input parameter, and the solid thermal conductivity will be the prediction results. The experimental data of the carbon aerogel elastic modulus are revisited as shown in Figure 2a. Figure 2b shows the comparison between the experimental data of the solid thermal conductivity and the calculations of Equation (4), where the experimental data were extracted from the measured effective thermal conductivity by the heat-transfer model of Equation (6). For low-density carbon aerogels, good agreements exist under *ρ* > 100 kg/m^3^, and the deviation increases as the density decreases. For high-density carbon aerogels, the predictions agree well with the experimental data.

From the above two validation examples of Equations (4) and (5), we can conclude that the thermal conduction model of the elastic modulus is suitable for the calculation of the solid thermal conductivity and elastic modulus of carbon aerogels, which is expected to be an alternative test method for the elastic modulus of high-density fragile aerogels.

### 2.2. Power-Scaling Model of Elastic Modulus of Carbon Aerogels

**Figure 3:**
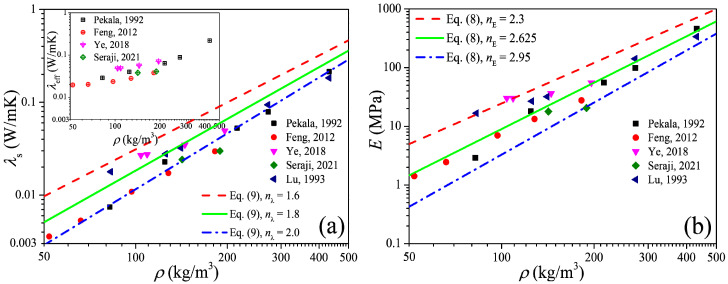
Elastic modulus of carbon aerogels converted from experimental thermal conductivity. (**a**) Thermal conductivity. (**b**) Elastic modulus.

The power-scaling model is a highly favored model to predict the physical properties of the porous material due to its simple form and convenience of calculation, and in this section we focus on the power-scaling exponent (i.e., *n*_E_ in Equation (8)) distribution interval as well as its median value by analysis of the available data of the elastic modulus of carbon aerogels. Figure 3a shows the open data [26,29,30,31,32] of the thermal conductivity of carbon aerogels, in which the data of the solid thermal conductivity (*λ*_s_) were extracted from the experimental data of the effective thermal conductivity (*λ*_eff_) by the heat-transfer model of Equation (6). Figure 3b shows the elastic modulus converted from the solid thermal conductivity by Equation (5). In the log–log coordinates, the elastic modulus increases approximately linearly with the increase in the density, and this indicates the power exponential function of Equation (8) can definitely describe the relation between the elastic modulus and the solid thermal conductivity. By fitting the data, the upper and lower limits as well as the median value of the exponent can be obtained, i.e., *n*_E_ ∈ [2.3, 2.95] with an arithmetic mean value of 2.625.

**Figure 4:**
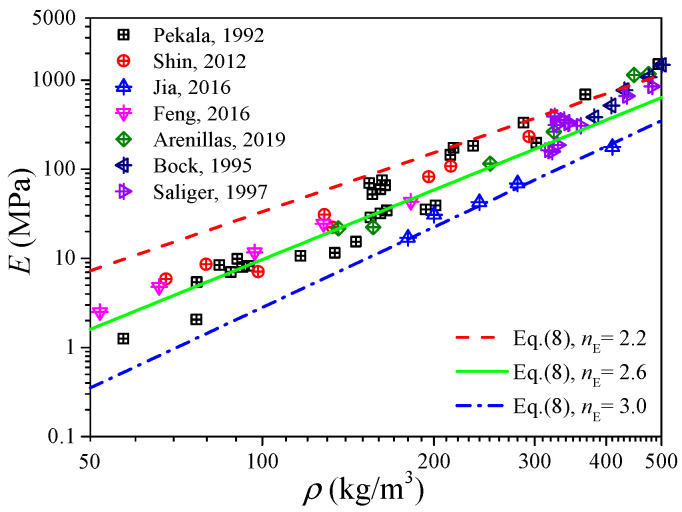
Experimental elastic modulus of carbon aerogel.

Figure 4 shows the measured data of the elastic modulus of the carbon aerogels in the literature [1,5,8,28,33,34,35], and an approximated linear relation between the elastic modulus and density can also be observed in the log–log coordinates. Fitting the data in Figure 4 as the form of Equation (8), we can obtain *n*_E_ ∈ [2.2, 3.0] with a mean value of 2.6, of which the distribution interval as well as the median value is very close to that obtained from Figure 3b. According to Figure 3b and Figure 4, we can conclude that the elastic modulus of carbon aerogels is mostly distributed in the range of *E*_0_(*ρ*/*ρ*_0_)^3.0^ ≤ *E* ≤ *E*_0_(*ρ*/*ρ*_0_)^2.2^, and the relation of *E* = *E*_0_(*ρ*/*ρ*_0_)^2.6^ can be used to estimate the elastic modulus for most carbon aerogels, such as the good predictions in Figure 1b.

### 2.3. Relation of Solid Thermal Conductivity and Elastic Modulus of Carbon Aerogels

To validate Equation (10), we substitute the median value of *n*_E_ = 2.6 obtained in Section 2.2 into Equation (10), and we have *n*_λ_ = 1.8. In Figure 1b and Figure 2b, good predictions also can be observed when *ρ* > 100 kg/m^3^, which is consistent with the results in Section 2.1. Furthermore, substituting *n*_E_ ∈ [2.2, 3.0] into Equation (10), we can obtain *n*_λ_ ∈ [1.6, 2.0], and in Figure 3a we can see that most of the experimental data of solid thermal conductivity are distributed between the predictions of *λ*_0_(*ρ*/*ρ*_0_)^2.0^ and *λ*_0_(*ρ*/*ρ*_0_)^1.6^, which also indicates that Equation (10) reflects the relationship between the elastic modulus and solid thermal conductivity of carbon aerogels and can be used to accurately predict the solid thermal conductivity of carbon aerogels.

### 2.4. Elastic Modulus of Carbon Nanotube Aerogels and Graphene Aerogels

Carbon nanotube aerogel (CNTA) and graphene aerogel (GA) are the two most interesting carbon-based aerogels due to their good electrical conductivity and their having superior mechanical properties to those of the traditional carbon aerogel (CA). The structure of CNTA or GA presents a three-dimensional network of CNTs or graphene instead of an amorphous assembly network consisting of carbon nanoparticles, and the primary CNT or graphene unit with crystalline nature enhances the mechanical properties of the network significantly compared with CA [2,3]. In this section, we focus on obtaining a scaling model as the form of Equation (8) for accurately predicting the elastic modulus of CNTA and GA. Similar to Section 2.2, the scaling exponent interval of CNTA and GA will also be derived by an analysis of available data in the literature, and then the median value of the upper and lower limits will be regarded as the value of the scaling exponent in Equation (8).

**Figure 5:**
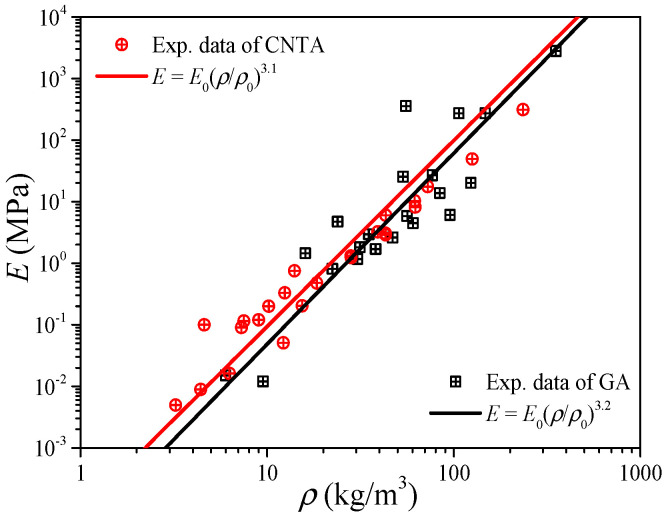
Elastic modulus of carbon-based aerogel.

**Figure 6:**
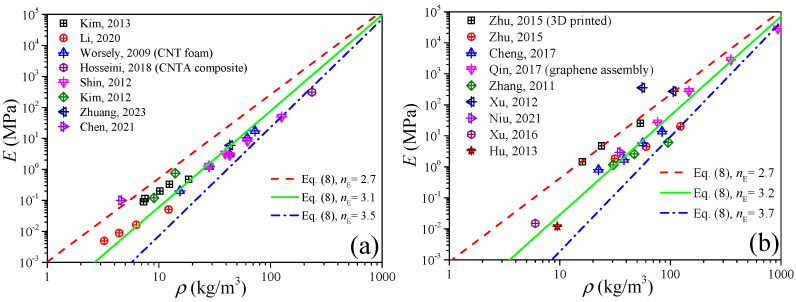
Elastic modulus of CNTA and GA. (**a**) CNTA. (**b**) GA.

Since the carbon nanotube can be viewed as a graphene sheet coiled into seamless tubes, and also their values of mechanical properties are very close (see Table 1), the CNTA and GA can be regarded as a class of materials from the view of elastic modulus predictions by Equation (8). Figure 5 shows the experimental data of the elastic modulus of the CNTA and GA in the literature [33,36,37,38,39,40,41,42,43,44,45,46,47,48,49,50], and the reference index is shown in Figure 6. By fitting the data in the form of Equation (8), like the CA, both the CNTA and GA present a linear relation between the density and elastic modulus in the log to log coordinates, and this proves that the power-scaling model also can be applicable to CNTA and GA. The relations of *E* = *E*_0_(*ρ*/*ρ*_0_)^3.1^ and *E* = *E*_0_(*ρ*/*ρ*_0_)^3.2^ may help us to predict the elastic modulus for the CNTA and GA, respectively.

If from the perspective of material dimension, CNT is the one-dimensional material while graphene is the two-dimensional material, we still need to distinguish them. Figure 6a,b plot the elastic modulus of CNTA and GA, respectively. In Figure 6a, we can obtain that the exponent of CNTA is *n*_E_ ∈ [2.7, 3.5], i.e., *E*_0_(*ρ*/*ρ*_0_)^3.5^ ≤ *E* ≤ *E*_0_(*ρ*/*ρ*_0_)^2.7^, while in Figure 6b we can observe that the elastic modulus of GA is distributed in the range of *E*_0_(*ρ*/*ρ*_0_)^3.7^ ≤ *E* ≤ *E*_0_(*ρ*/*ρ*_0_)^2.7^.

In addition, for low density, the predictions in Figure 6 are in good agreement with the experimental data, which is different from the deviations in Figure 1 and Figure 2, and this is because the bulk sound velocity of CNT and graphene is much higher than that of air, and under this condition the density effect will be less important than that of the sound velocity.

## 3. Conclusions

In this study, we investigated the elastic modulus of the carbon-based aerogels, and focused on improving the prediction accuracy of the power-scaling model for the elastic modulus of carbon-based aerogels. We set the prefactor of the Gibson and Ashby equation as *C* = 1.0, and then the key to improving the accuracy lies in obtaining an accurate exponent. Available experimental data in the literature were employed to fit relations as the form of the power-scaling model, and the exponent of carbon aerogels is mostly distributed in the range from 2.2 to 3.0, which is much narrower that the range of 2.0–4.0 reported in the literature. The median value of 2.6 was recommended as the exponent of the power-scaling model to predict the elastic modulus of carbon aerogels. The thermal conduction model of the elastic modulus of silica aerogels was also employed to predict the carbon aerogel elastic modulus, and the predictions agreed well with the experimental data, which proved that the thermal conduction model of elastic modulus is also suitable for the calculation of the solid thermal conductivity and elastic modulus of carbon aerogels. The relation between the solid thermal conductivity and elastic modulus of carbon aerogels was established in the form of a power-scaling model, and the exponents of solid thermal conductivity and elastic modulus can be expressed as *n*_λ_ = (*n*_E_ + 1)/2. The elastic modulus of the carbon nanotube and graphene aerogels as two allotropes of carbon aerogel was also discussed. Similar to the carbon aerogels, we still prefer the power-scaling model of the elastic modulus. The distribution intervals of carbon nanotube aerogels and graphene aerogels were about [2.7, 3.5] and [2.7, 3.7] fitted from the open experimental data, and the median values of 3.1 and 3.2 are recommended as the exponents in the scaling model to calculate the elastic modulus of the two types of carbon-based aerogels, respectively.

## 4. Materials and Methods

### 4.1. Definition of Elastic Modulus

Elastic moduli of solid materials include Yang’s modulus (*E*), shear modulus (*G*), and bulk modulus (*K*). In this study, the elastic modulus specifically refers to Young’s modulus, since the shear modulus and the bulk modulus can be deduced from Yang’s modulus [51]. The definition of *E* is the slope of the stress–strain curve in the elastic deformation during the mechanical tensile or compression test, i.e., *σ* = *E*·*ε*, where *σ* is the stress, and *ε* the elastic strain in the direction of principal stress. Due to the brittleness of the carbon aerogel, the elastic modulus is mostly measured by the mechanical compression test.

### 4.2. Elastic Mechanical Model of Elastic Modulus

Due to the high brittleness of carbon aerogels, the mechanical compression test of the elastic modulus may easily lead to some damage to the structure of the material, and thus large measurement errors may be brought in. An alternative method is to measure the sound velocity in the material, and then use the elastic mechanical model to calculate the elastic modulus, expressed by [52],

(1) $$E=\frac{\rho v_{l}^{2}(1+\mu )(1-2\mu )}{\left(1-\mu \right)}=2\rho v_{t}^{2}(1+\mu )$$

(2) $$\mu =\frac{1-2{\left(v_{t}/v_{l}\right)}^{2}}{2-2{\left(v_{t}/v_{l}\right)}^{2}}$$

where *v*_l_ is the longitudinal sound velocity, *v*_t_ is the transverse sound velocity, *ρ* is the density, and *μ* is the Poisson’s ratio of the material. The sound velocity in aerogels can be measured by the ultrasonic echo method and the Brillouin scattering method [51].

### 4.3. Thermal Conduction Model of Elastic Modulus

According to the author’s previous work [25], the bridge between the elastic modulus and solid thermal conductivity of the silica aerogel is the sound velocity, and combining the scaling sound velocity model of the solid thermal conductivity [53],

(3) $$\lambda_{s}=\lambda_{0}\frac{\rho }{\rho_{0}}\frac{v}{v_{0}}$$

with the elastic mechanical model Equations (1) and (2), we yield

(4) $$\lambda_{s}=\lambda_{0}\frac{\rho }{\rho_{0}}\frac{1}{v_{0}}\left(\frac{1}{3}\sqrt{\frac{E(1-\mu )}{\rho (1+\mu )(1-2\mu )}}+\frac{2}{3}\sqrt{\frac{E}{2\rho (1+\mu )}}\right)$$

and

(5) $$E=2\rho (1+\mu )v_{0}^{2}\left[\frac{9\lambda_{s}^{2}\rho_{0}^{2}}{\lambda_{0}^{2}\rho^{2}}\cdot \frac{1}{{\left(\sqrt{\left(2-2\mu )/(1-2\mu \right)}+2\right)}^{2}}\right]$$

where *v* is the average sound velocity approximated by *v* = 1/3*v*_l_ + 2/3*v*_t_, *v*_0_ is the average sound velocity in the dense solid, *λ*_0_ is the thermal conductivity of the aerogel solid backbone (~the bulk value of the dense solid), and *λ*_s_ is the solid thermal conductivity which is the contribution of the thermal conductivity of the aerogel solid backbone to the effective thermal conductivity of the aerogel. Note that Equations (4) and (5) are the inverse operations between the elastic modulus and solid thermal conductivity. The solid thermal conductivity can be obtained by subtracting the gaseous thermal conductivity (*λ*_g_), radiative thermal conductivity (*λ*_r_), and coupling thermal conductivity (*λ*_c_) from the effective thermal conductivity (*λ*_eff_) of the aerogel [7],

(6) $$\lambda_{s}=\lambda_{\mathrm{eff}}-\lambda_{g}-\lambda_{c}-\lambda_{r}$$

where *λ*_eff_ can be directly measured by the thermal conductivity equipment, and *λ*_g_, *λ*_c_, and *λ*_r_ can be calculated by the heat transfer models. The gaseous thermal conductivity approximates to *λ*_g_ ≈ *Cλ*_g,free_/(1 + 2*βΛ*_g_/*D*), where *λ*_g,free_ is the thermal conductivity of the gas in free space, *Λ*_g_ is the mean free path of the gas molecules filled in the aerogel, *D* is the mean pore diameter of the aerogel (for carbon aerogels, *D* ≈ 333 exp(−*ρ*/86.8) + 37 fitted from the experimental data in Ref. [29]), *β* is a gas-dependent coefficient with *β* = 1.55 for air, and *C* is a correction coefficient with the consideration of the non-uniform pore size distribution effect, which is often in the range of 1.1–1.2 and one can use *C* = 1.15 for simple calculations. The coupling thermal conductivity can be accurately calculated by *λ*_c_ = 2/3*λ*_g_ when *λ*_0_ >> *λ*_g_, such as silica aerogel, carbon aerogel, and alumina aerogel. The radiative thermal conductivity is as the functions of the density and temperature described by the photon-diffusion model, i.e., *λ*_g_ = 16*σ*_B_*T*^3^/(3*ρe*_s_), where *σ*_B_ is the Stefan–Boltzmann constant, and *e*_s_ is the specific Rossland mean extinction coefficient (for carbon aerogels, *e*_s_ ≈ 146 m^3^/kg [54]). Note that once the effective thermal conductivity of the aerogel is measured in a vacuum (*p* < 10 Pa) and at room temperature, the measured result can be regarded as the solid thermal conductivity. Although the thermal conduction model of elastic modulus was proposed based on the structure of silica aerogel, i.e., amorphous porous structure of silica particle assembly, it is still applicable to the traditional carbon aerogel due to its amorphous particle assembly porous structure, and the validation is described in Section 2.1.

### 4.4. Power-Scaling Model of Elastic Modulus of Porous Material

The power-scaling model of porous material was proposed by Gibson and Ashby in 1982 [20], and has been widely used to estimate many properties of porous materials due to its simple expression, given by,

(7) $$\frac{x}{x_{0}}=C{\left(\frac{\rho }{\rho_{0}}\right)}^{n}$$

where *C* is a constant (prefactor), *ρ* is the density of the porous material, *x* is the expected physical property, and subscript “0” denotes the bulk value of the dense solid. Equation (7) was proposed based on the foam structure modeling, and is applicable to the relative density of 0.01–1.0 [20], but it has also been proved to be applicable to amorphous porous carbon-based aerogels [36,48]. For the elastic modulus, when the density of the carbon aerogel approaches that of the dense carbon solid in theory, i.e., *ρ*/*ρ*_0_ = 1, which can be regarded as the boundary condition of Equation (7), we can believe that the elastic modulus of the aerogel is equal to the bulk value. Then, we can obtain *C* = 1.0 in Equation (7) and the elastic modulus can be written as

(8) $$\frac{E}{E_{0}}={\left(\frac{\rho }{\rho_{0}}\right)}^{n_{E}}$$

where *n*_E_ is the power-scaling exponent of the elastic modulus which ranges from 2–4 for aerogels [51]. Note that, once the density of the aerogel is too low to neglect the effect of the air elasticity on the elastic modulus, we would obtain *E* > 0 and *C* ≠ 1 under the condition of *ñ* = 0. In the present study, *C* = 1 can be used for carbon aerogel under *ñ* > 100 kg/m^3^ as observed in Figure 1 and Figure 2. For CNTA and GA, the bulk sound velocity of CNT and graphene is much higher than that of the air, and under this condition the density effect will be less important than that of the sound velocity, so *C* = 1 can also be used for CNTA and GA, which is also proved in Refs. [36,48]. In addition, the range of *n*_E_ is relatively wide, and this is not conducive to obtaining accurate predictions for the elastic modulus of carbon aerogels. For this reason, we prefer to narrow the range of the *n*_E_ based on the analysis of the open data in the literature, and then take a reasonable mean value to obtain more accurate predictions.

### 4.5. Power-Scaling Model of Solid Thermal Conductivity of Carbon Aerogels

As mentioned in Section 4.3, the elastic modulus and solid thermal conductivity of aerogels can be predicted from each other under a given density. Once we obtain the scaling model of the elastic modulus of carbon aerogels, we also expect a scaling model to simplify the calculations of the solid thermal conductivity of carbon aerogels, and the scaling model based on the expression of Equation (7) can be written as below,

(9) $$\frac{\lambda_{s}}{\lambda_{0}}={\left(\frac{\rho }{\rho_{0}}\right)}^{n_{\lambda }}$$

where *n*_λ_ is the scaling exponent of the solid thermal conductivity of carbon aerogels. The parameter *n*_λ_ can be deduced from *n*_E_ by solving Equations (5), (8), and (9), and the simple derivation process is as follows: (1) Equations (5), (8), and (9) can be regarded as $E\propto {\lambda_{s}}^{2}/\rho$, $E\propto \rho^{n_{E}}$, and $\lambda_{s}\propto \rho^{n_{\lambda }}$, respectively; (2) making Equation (5) equal to Equation (8), we can obtain ${\lambda_{s}}^{2}\propto \rho^{n_{E}+1}$; and (3) comparing $\lambda_{s}\propto \rho^{n_{\lambda }}$ with ${\lambda_{s}}^{2}\propto \rho^{n_{E}+1}$, the scaling exponent *n*_λ_ can be expressed as,

(10) $$n_{\lambda }=\frac{n_{E}+1}{2}$$

### 4.6. Bulk Properties of Carbon Allotropes

Before the calculations of the carbon aerogel elastic modulus, the bulk properties of the dense carbon are necessary according to Equations (4), (5), and (8). In this work, the properties of amorphous carbon, single wall carbon nanotube, and monolayer graphene are used for the bulk values of the carbon aerogel, carbon nanotube aerogel, and graphene aerogel, respectively, and the related properties under room temperature are listed in Table 1. Note that, (1) for amorphous carbon, the values of density and thermal conductivity are taken from graphite [55], the elastic modulus adopts the value of glassy carbon [56], the average sound velocity is cited from Ref. [57], and the values of the longitude and transverse sound velocity are calculated by *v* = 1/3*v*_l_ + 2/3*v*_t_ with the approximation of *v*_t_ ≈ 0.6*v*_l_; (2) for carbon nanotube, the value of the density takes the isolated single wall carbon nanotube [58], the elastic modulus is cited from Ref. [59], the thermal conductivity is from Ref. [60], and the sound velocities are the theoretical calculations in Ref. [61]; and (3) for the graphene, the elastic modulus and density are taken from Ref. [36], the thermal conductivity is from Ref. [62], and the sound velocities are the experimental data published in Ref. [63].

## Figures and Tables

**Table 1:** Bulk properties for prediction of elastic modulus of carbon-based aerogels.

Carbon Allotropes	*ρ*_0_ (kg/m^3^)	*E*_0_ (MPa)	*λ*_0_ (W/mK)	(*v*_t_)_0_ (km/s)	(*v*_l_)_0_ (km/s)	*v*_0_ (km/s)
Amorphous carbon	2200	30,000	5.6	3.27	5.45	4.0
Carbon nanotube	2100	970,000	2000–6000	9.43	20.35	—
Graphene	2300	1,020,000	5000	12.9	19.9	—

## Data Availability

The original contributions presented in this study are included in the article. Further inquiries can be directed to the corresponding authors.
